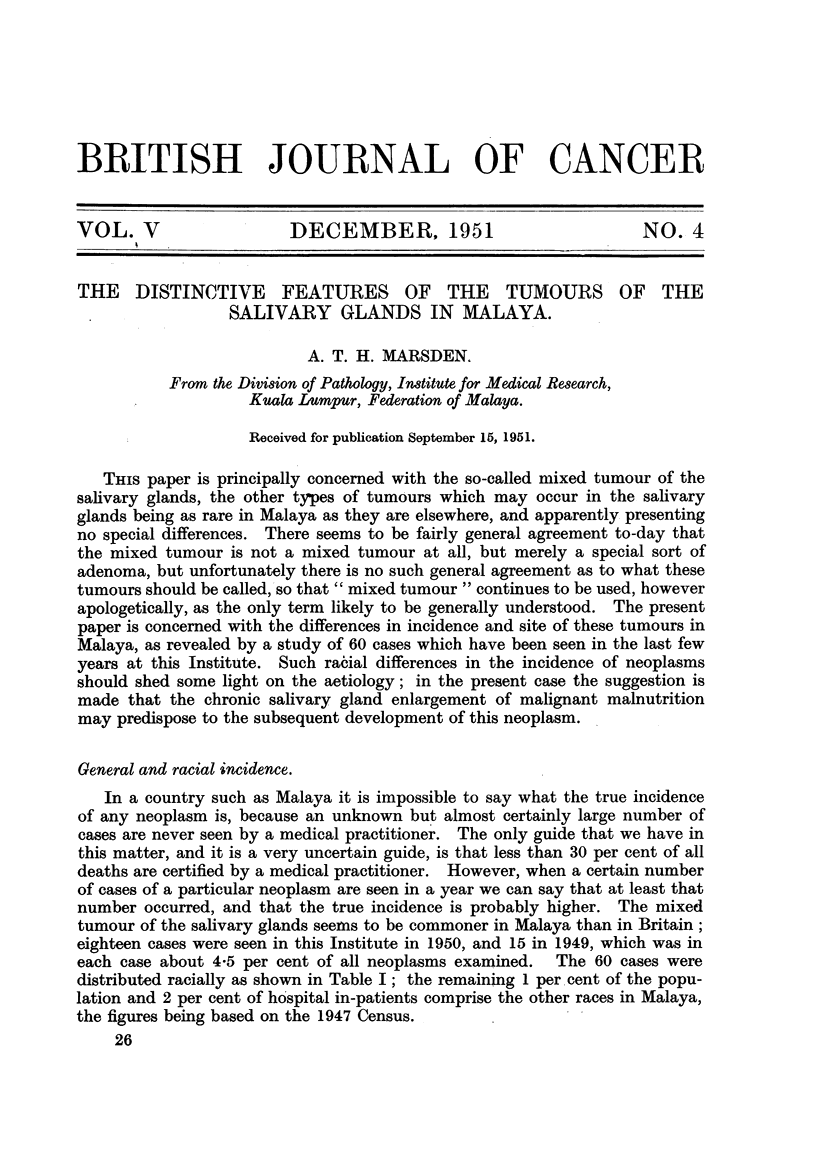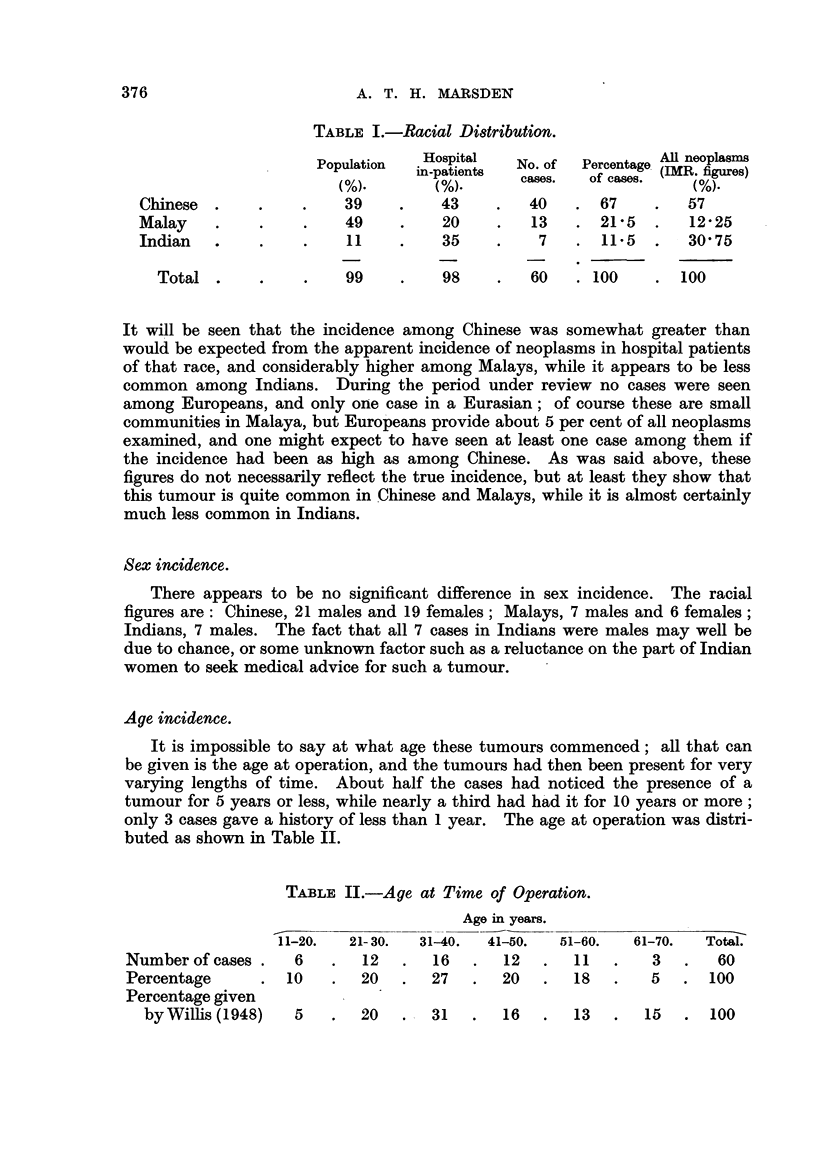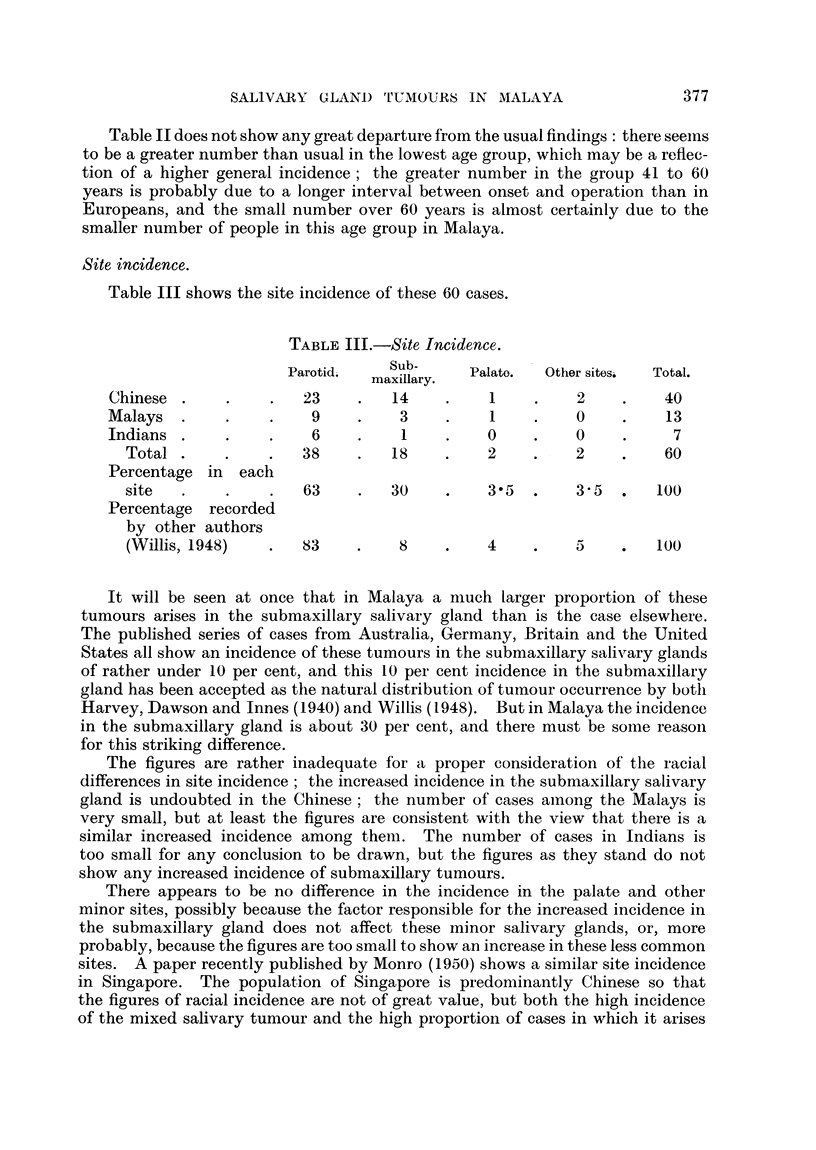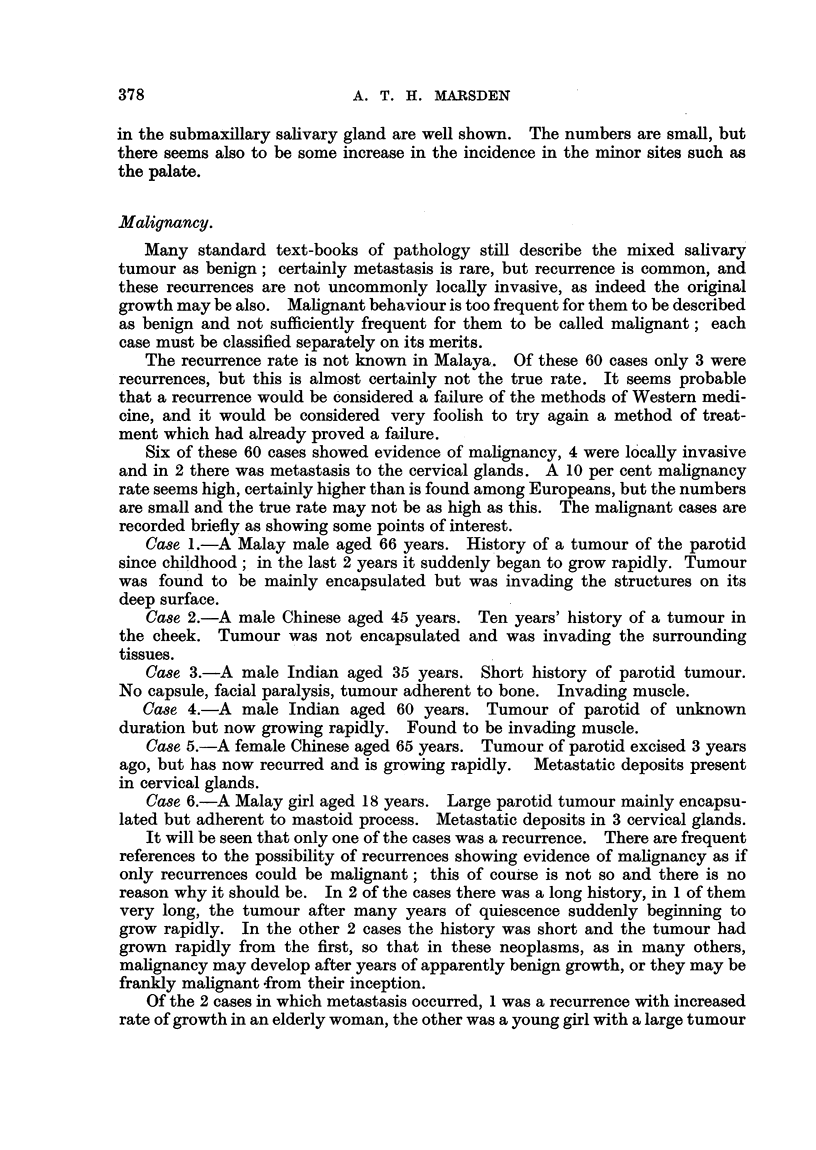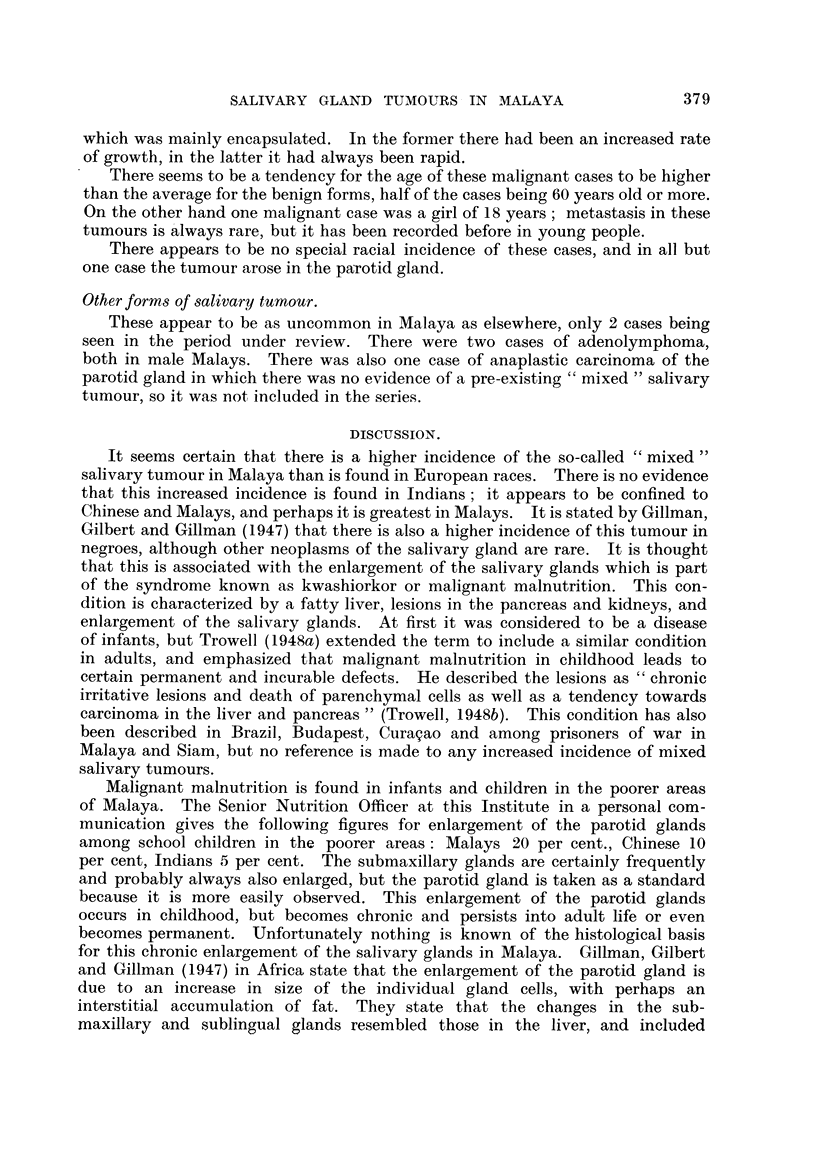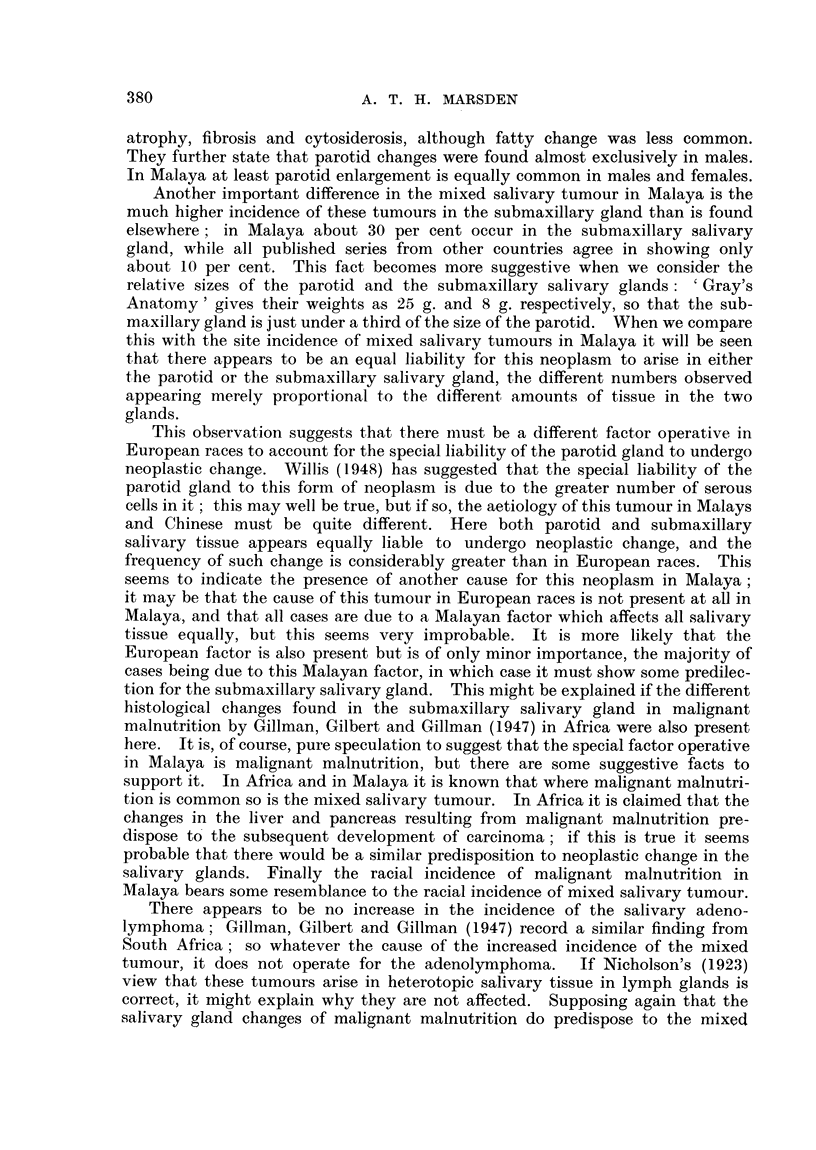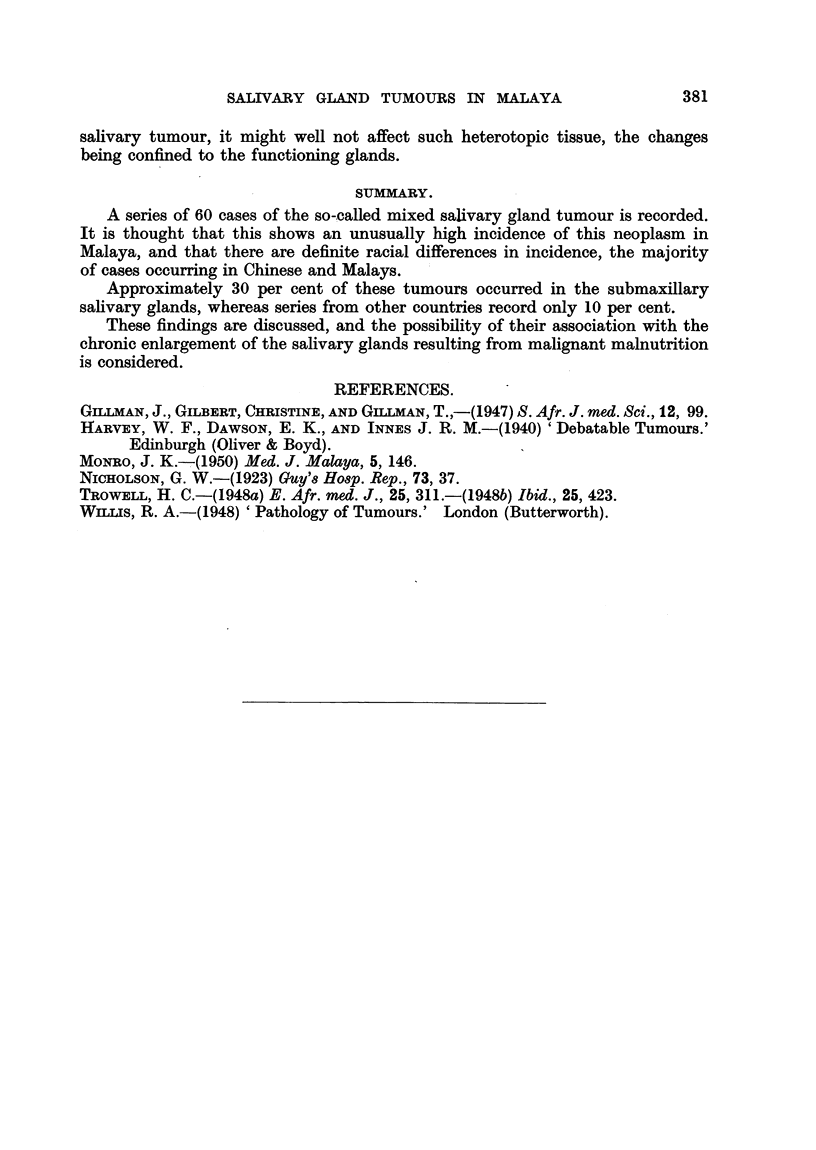# The Distinctive Features of the Tumours of the Salivary Glands in Malaya

**DOI:** 10.1038/bjc.1951.42

**Published:** 1951-12

**Authors:** A. T. H. Marsden


### BRITISH JOURNAL OF CANCER

VOL. V DECEMBER, 1951 NO. 4

### THE DISTINCTIVE FEATURES OF THE TUMOURS OF THE SALIVARY GLANDS IN MALAYA.

A. T. H. MARSDEN.

*From the Division of Pathology, Institute for Medical Research, Kuala Lumpur, Federation of Malaya.*

Received for publication September 15, 1951.

THIS paper is principally concerned with the so-called mixed tumour of the salivary glands, the other types of tumours which may occur in the salivary glands being as rare in Malaya as they are elsewhere, and apparently presenting no special differences. There seems to be fairly general agreement to-day that the mixed tumour is not a mixed tumour at all, but merely a special sort of adenoma, but unfortunately there is no such general agreement as to what these tumours should be called, so that " mixed tumour " continues to be used, however apologetically, as the only term likely to be generally understood. The present paper is concerned with the differences in incidence and site of these tumours in Malaya, as revealed by a study of 60 cases which have been seen in the last few years at this Institute. Such racial differences in the incidence of neoplasms should shed some light on the aetiology ; in the present case the suggestion is made that the chronic salivary gland enlargement of malignant malnutrition may predispose to the subsequent development of this neoplasm.

*General and racial incidence.*

In a country such as Malaya it is impossible to say what the true incidence of any neoplasm is, because an unknown but almost certainly large number of cases are never seen by a medical practitioner. The only guide that we have in this matter, and it is a very uncertain guide, is that less than 30 per cent of all deaths are certified by a medical practitioner. However, when a certain number of cases of a particular neoplasm are seen in a year we can say that at least that number occurred, and that the true incidence is probably higher. The mixed tumour of the salivary glands seems to be commoner in Malaya than in Britain ; eighteen cases were seen in this Institute in 1950, and 15 in 1949, which was in each case about 4·5 per cent of all neoplasms examined. The 60 cases were distributed racially as shown in Table I ; the remaining 1 per cent of the population and 2 per cent of hospital in-patients comprise the other races in Malaya, the figures being based on the 1947 Census.

TABLE I.—*Racial Distribution.*

| | Population (%). | Hospital in-patients (%). | No. of cases. | Percentage of cases. | All neoplasms (IMR. figures) (%). |
|---|---|---|---|---|---|
| Chinese | 39 | 43 | 40 | 67 | 57 |
| Malay | 49 | 20 | 13 | 21·5 | 12·25 |
| Indian | 11 | 35 | 7 | 11·5 | 30·75 |
| Total | 99 | 98 | 60 | 100 | 100 |

It will be seen that the incidence among Chinese was somewhat greater than would be expected from the apparent incidence of neoplasms in hospital patients of that race, and considerably higher among Malays, while it appears to be less common among Indians. During the period under review no cases were seen among Europeans, and only one case in a Eurasian; of course these are small communities in Malaya, but Europeans provide about 5 per cent of all neoplasms examined, and one might expect to have seen at least one case among them if the incidence had been as high as among Chinese. As was said above, these figures do not necessarily reflect the true incidence, but at least they show that this tumour is quite common in Chinese and Malays, while it is almost certainly much less common in Indians.

*Sex incidence.*

There appears to be no significant difference in sex incidence. The racial figures are: Chinese, 21 males and 19 females; Malays, 7 males and 6 females; Indians, 7 males. The fact that all 7 cases in Indians were males may well be due to chance, or some unknown factor such as a reluctance on the part of Indian women to seek medical advice for such a tumour.

*Age incidence.*

It is impossible to say at what age these tumours commenced; all that can be given is the age at operation, and the tumours had then been present for very varying lengths of time. About half the cases had noticed the presence of a tumour for 5 years or less, while nearly a third had had it for 10 years or more; only 3 cases gave a history of less than 1 year. The age at operation was distributed as shown in Table II.

TABLE II.—*Age at Time of Operation.*

| | Age in years. | | | | | | |
|---|---|---|---|---|---|---|---|
| | 11–20. | 21-30. | 31–40. | 41–50. | 51–60. | 61–70. | Total. |
| Number of cases | 6 | 12 | 16 | 12 | 11 | 3 | 60 |
| Percentage | 10 | 20 | 27 | 20 | 18 | 5 | 100 |
| Percentage given by Willis (1948) | 5 | 20 | 31 | 16 | 13 | 15 | 100 |

Table II does not show any great departure from the usual findings : there seems to be a greater number than usual in the lowest age group, which may be a reflection of a higher general incidence ; the greater number in the group 41 to 60 years is probably due to a longer interval between onset and operation than in Europeans, and the small number over 60 years is almost certainly due to the smaller number of people in this age group in Malaya.

*Site incidence.*

Table III shows the site incidence of these 60 cases.

TABLE III.—*Site Incidence.*

| | Parotid. | Sub-maxillary. | Palate. | Other sites. | Total. |
|---|---|---|---|---|---|
| Chinese | 23 | 14 | 1 | 2 | 40 |
| Malays | 9 | 3 | 1 | 0 | 13 |
| Indians | 6 | 1 | 0 | 0 | 7 |
| Total | 38 | 18 | 2 | 2 | 60 |
| Percentage in each site | 63 | 30 | 3·5 | 3·5 | 100 |
| Percentage recorded by other authors (Willis, 1948) | 83 | 8 | 4 | 5 | 100 |

It will be seen at once that in Malaya a much larger proportion of these tumours arises in the submaxillary salivary gland than is the case elsewhere. The published series of cases from Australia, Germany, Britain and the United States all show an incidence of these tumours in the submaxillary salivary glands of rather under 10 per cent, and this 10 per cent incidence in the submaxillary gland has been accepted as the natural distribution of tumour occurrence by both Harvey, Dawson and Innes (1940) and Willis (1948). But in Malaya the incidence in the submaxillary gland is about 30 per cent, and there must be some reason for this striking difference.

The figures are rather inadequate for a proper consideration of the racial differences in site incidence ; the increased incidence in the submaxillary salivary gland is undoubted in the Chinese ; the number of cases among the Malays is very small, but at least the figures are consistent with the view that there is a similar increased incidence among them. The number of cases in Indians is too small for any conclusion to be drawn, but the figures as they stand do not show any increased incidence of submaxillary tumours.

There appears to be no difference in the incidence in the palate and other minor sites, possibly because the factor responsible for the increased incidence in the submaxillary gland does not affect these minor salivary glands, or, more probably, because the figures are too small to show an increase in these less common sites. A paper recently published by Monro (1950) shows a similar site incidence in Singapore. The population of Singapore is predominantly Chinese so that the figures of racial incidence are not of great value, but both the high incidence of the mixed salivary tumour and the high proportion of cases in which it arises in the submaxillary salivary gland are well shown. The numbers are small, but there seems also to be some increase in the incidence in the minor sites such as the palate.

*Malignancy.*

Many standard text-books of pathology still describe the mixed salivary tumour as benign; certainly metastasis is rare, but recurrence is common, and these recurrences are not uncommonly locally invasive, as indeed the original growth may be also. Malignant behaviour is too frequent for them to be described as benign and not sufficiently frequent for them to be called malignant; each case must be classified separately on its merits.

The recurrence rate is not known in Malaya. Of these 60 cases only 3 were recurrences, but this is almost certainly not the true rate. It seems probable that a recurrence would be considered a failure of the methods of Western medicine, and it would be considered very foolish to try again a method of treatment which had already proved a failure.

Six of these 60 cases showed evidence of malignancy, 4 were locally invasive and in 2 there was metastasis to the cervical glands. A 10 per cent malignancy rate seems high, certainly higher than is found among Europeans, but the numbers are small and the true rate may not be as high as this. The malignant cases are recorded briefly as showing some points of interest.

*Case* 1.—A Malay male aged 66 years. History of a tumour of the parotid since childhood; in the last 2 years it suddenly began to grow rapidly. Tumour was found to be mainly encapsulated but was invading the structures on its deep surface.

*Case* 2.—A male Chinese aged 45 years. Ten years' history of a tumour in the cheek. Tumour was not encapsulated and was invading the surrounding tissues.

*Case* 3.—A male Indian aged 35 years. Short history of parotid tumour. No capsule, facial paralysis, tumour adherent to bone. Invading muscle.

*Case* 4.—A male Indian aged 60 years. Tumour of parotid of unknown duration but now growing rapidly. Found to be invading muscle.

*Case* 5.—A female Chinese aged 65 years. Tumour of parotid excised 3 years ago, but has now recurred and is growing rapidly. Metastatic deposits present in cervical glands.

*Case* 6.—A Malay girl aged 18 years. Large parotid tumour mainly encapsulated but adherent to mastoid process. Metastatic deposits in 3 cervical glands.

It will be seen that only one of the cases was a recurrence. There are frequent references to the possibility of recurrences showing evidence of malignancy as if only recurrences could be malignant; this of course is not so and there is no reason why it should be. In 2 of the cases there was a long history, in 1 of them very long, the tumour after many years of quiescence suddenly beginning to grow rapidly. In the other 2 cases the history was short and the tumour had grown rapidly from the first, so that in these neoplasms, as in many others, malignancy may develop after years of apparently benign growth, or they may be frankly malignant from their inception.

Of the 2 cases in which metastasis occurred, 1 was a recurrence with increased rate of growth in an elderly woman, the other was a young girl with a large tumour which was mainly encapsulated. In the former there had been an increased rate of growth, in the latter it had always been rapid.

There seems to be a tendency for the age of these malignant cases to be higher than the average for the benign forms, half of the cases being 60 years old or more. On the other hand one malignant case was a girl of 18 years ; metastasis in these tumours is always rare, but it has been recorded before in young people.

There appears to be no special racial incidence of these cases, and in all but one case the tumour arose in the parotid gland.

*Other forms of salivary tumour.*

These appear to be as uncommon in Malaya as elsewhere, only 2 cases being seen in the period under review. There were two cases of adenolymphoma, both in male Malays. There was also one case of anaplastic carcinoma of the parotid gland in which there was no evidence of a pre-existing " mixed " salivary tumour, so it was not included in the series.

### DISCUSSION.

It seems certain that there is a higher incidence of the so-called " mixed " salivary tumour in Malaya than is found in European races. There is no evidence that this increased incidence is found in Indians ; it appears to be confined to Chinese and Malays, and perhaps it is greatest in Malays. It is stated by Gillman, Gilbert and Gillman (1947) that there is also a higher incidence of this tumour in negroes, although other neoplasms of the salivary gland are rare. It is thought that this is associated with the enlargement of the salivary glands which is part of the syndrome known as kwashiorkor or malignant malnutrition. This condition is characterized by a fatty liver, lesions in the pancreas and kidneys, and enlargement of the salivary glands. At first it was considered to be a disease of infants, but Trowell (1948*a*) extended the term to include a similar condition in adults, and emphasized that malignant malnutrition in childhood leads to certain permanent and incurable defects. He described the lesions as " chronic irritative lesions and death of parenchymal cells as well as a tendency towards carcinoma in the liver and pancreas " (Trowell, 1948*b*). This condition has also been described in Brazil, Budapest, Curaçao and among prisoners of war in Malaya and Siam, but no reference is made to any increased incidence of mixed salivary tumours.

Malignant malnutrition is found in infants and children in the poorer areas of Malaya. The Senior Nutrition Officer at this Institute in a personal communication gives the following figures for enlargement of the parotid glands among school children in the poorer areas : Malays 20 per cent., Chinese 10 per cent, Indians 5 per cent. The submaxillary glands are certainly frequently and probably always also enlarged, but the parotid gland is taken as a standard because it is more easily observed. This enlargement of the parotid glands occurs in childhood, but becomes chronic and persists into adult life or even becomes permanent. Unfortunately nothing is known of the histological basis for this chronic enlargement of the salivary glands in Malaya. Gillman, Gilbert and Gillman (1947) in Africa state that the enlargement of the parotid gland is due to an increase in size of the individual gland cells, with perhaps an interstitial accumulation of fat. They state that the changes in the submaxillary and sublingual glands resembled those in the liver, and included atrophy, fibrosis and cytosiderosis, although fatty change was less common. They further state that parotid changes were found almost exclusively in males. In Malaya at least parotid enlargement is equally common in males and females.

Another important difference in the mixed salivary tumour in Malaya is the much higher incidence of these tumours in the submaxillary gland than is found elsewhere ; in Malaya about 30 per cent occur in the submaxillary salivary gland, while all published series from other countries agree in showing only about 10 per cent. This fact becomes more suggestive when we consider the relative sizes of the parotid and the submaxillary salivary glands : ‘ Gray’s Anatomy ’ gives their weights as 25 g. and 8 g. respectively, so that the submaxillary gland is just under a third of the size of the parotid. When we compare this with the site incidence of mixed salivary tumours in Malaya it will be seen that there appears to be an equal liability for this neoplasm to arise in either the parotid or the submaxillary salivary gland, the different numbers observed appearing merely proportional to the different amounts of tissue in the two glands.

This observation suggests that there must be a different factor operative in European races to account for the special liability of the parotid gland to undergo neoplastic change. Willis (1948) has suggested that the special liability of the parotid gland to this form of neoplasm is due to the greater number of serous cells in it ; this may well be true, but if so, the aetiology of this tumour in Malays and Chinese must be quite different. Here both parotid and submaxillary salivary tissue appears equally liable to undergo neoplastic change, and the frequency of such change is considerably greater than in European races. This seems to indicate the presence of another cause for this neoplasm in Malaya ; it may be that the cause of this tumour in European races is not present at all in Malaya, and that all cases are due to a Malayan factor which affects all salivary tissue equally, but this seems very improbable. It is more likely that the European factor is also present but is of only minor importance, the majority of cases being due to this Malayan factor, in which case it must show some predilection for the submaxillary salivary gland. This might be explained if the different histological changes found in the submaxillary salivary gland in malignant malnutrition by Gillman, Gilbert and Gillman (1947) in Africa were also present here. It is, of course, pure speculation to suggest that the special factor operative in Malaya is malignant malnutrition, but there are some suggestive facts to support it. In Africa and in Malaya it is known that where malignant malnutrition is common so is the mixed salivary tumour. In Africa it is claimed that the changes in the liver and pancreas resulting from malignant malnutrition predispose to the subsequent development of carcinoma ; if this is true it seems probable that there would be a similar predisposition to neoplastic change in the salivary glands. Finally the racial incidence of malignant malnutrition in Malaya bears some resemblance to the racial incidence of mixed salivary tumour.

There appears to be no increase in the incidence of the salivary adenolymphoma ; Gillman, Gilbert and Gillman (1947) record a similar finding from South Africa ; so whatever the cause of the increased incidence of the mixed tumour, it does not operate for the adenolymphoma. If Nicholson’s (1923) view that these tumours arise in heterotopic salivary tissue in lymph glands is correct, it might explain why they are not affected. Supposing again that the salivary gland changes of malignant malnutrition do predispose to the mixed salivary tumour, it might well not affect such heterotopic tissue, the changes being confined to the functioning glands.

SUMMARY.

A series of 60 cases of the so-called mixed salivary gland tumour is recorded. It is thought that this shows an unusually high incidence of this neoplasm in Malaya, and that there are definite racial differences in incidence, the majority of cases occurring in Chinese and Malays.

Approximately 30 per cent of these tumours occurred in the submaxillary salivary glands, whereas series from other countries record only 10 per cent.

These findings are discussed, and the possibility of their association with the chronic enlargement of the salivary glands resulting from malignant malnutrition is considered.

### REFERENCES.

GILLMAN, J., GILBERT, CHRISTINE, AND GILLMAN, T.,—(1947) *S. Afr. J. med. Sci.*, **12**, 99.

HARVEY, W. F., DAWSON, E. K., AND INNES J. R. M.—(1940) 'Debatable Tumours.' Edinburgh (Oliver & Boyd).

MONRO, J. K.—(1950) *Med. J. Malaya*, **5**, 146.

NICHOLSON, G. W.—(1923) *Guy's Hosp. Rep.*, **73**, 37.

TROWELL, H. C.—(1948*a*) *E. Afr. med. J.*, **25**, 311.—(1948*b*) *Ibid.*, **25**, 423.

WILLIS, R. A.—(1948) 'Pathology of Tumours.' London (Butterworth).